# Exogenous SDF-1α Protects Human Myocardium from Hypoxia-Reoxygenation Injury via CXCR4

**DOI:** 10.1007/s10557-015-6622-5

**Published:** 2015-10-20

**Authors:** A. Malik, D. I. Bromage, Z. He, L. Candilio, A. Hamarneh, S. Taferner, S. M. Davidson, D. M. Yellon

**Affiliations:** The Hatter Cardiovascular Institute, University College London, 67 Chenies Mews, London, WC1E 6HX UK

**Keywords:** SDF-1α, Stromal derived factor, CXCR4, Ischaemia-reperfusion injury, Cardioprotection

## Introduction

ST-segment elevation myocardial infarction (STEMI) is a consequence of atherosclerotic plaque rupture and thrombotic occlusion of the coronary artery causing downstream ischaemia and, eventually, cell death. The most effective therapeutic strategy for STEMI is timely reperfusion by primary percutaneous coronary intervention (PPCI). Such reperfusion is a prerequisite for myocardial salvage, leading to smaller infarct sizes and improved clinical outcomes [1, 2]. However, reperfusion can itself inflict further injury, termed ischaemia-reperfusion injury (IRI). Despite PPCI, a recent study found 30-day, 1-year, and 5-year mortality following STEMI to be 7.9 %, 11.4 %, and 23.3 %, respectively [3]. Consequently, novel strategies to mitigate the deleterious effects of IRI are paramount.

Stromal derived factor-1α (SDF-1α or CXCL12) is a chemokine that has demonstrated cardioprotective activity in mice [4]. We recently demonstrated that exogenous SDF-1α improved functional recovery of ex vivo rat cardiac papillary muscle subjected to hypoxia and reoxygenation (simulated IRI) [5]. This effect was abrogated by pre-treatment with AMD3100, a highly specific antagonist of the SDF-1α receptor, CXCR4.

However, it is not known whether SDF-1α can similarly protect human heart tissue and whether any such protection is afforded via CXCR4. We address this question using isolated human atrial trabeculae subjected to simulated IRI.

## Methods

### Human Atrial Trabeculae Hypoxia-Reoxygenation Experiments

The study received Local Research Ethics Committee approval and was carried out in accordance with the University College London Hospitals NHS Trust guidelines. A right atrial appendage sample was harvested from 47 patients with chronic stable angina undergoing cannulation for cardiopulmonary bypass for CABG. All patients were aged 18–80 years and there were no significant differences in their baseline characteristics **(see** Table 1**)**. Patients with diabetes, impaired renal or ventricular function, dilated left atria, unstable angina, or a history of arrhythmias or on rhythm stabilising medications were excluded.

**Table 1:** Patient baseline characteristics

	Control (*n* = 11)	Hypoxic preconditioning (*n* = 10)	SDF-1α (*n* = 11)	SDF-1α + AMD3100 (*n* = 10)	AMD3100 (*n* = 5)
Mean age (years)	61	64.8	64.6	63.4	61.6
Gender (male)	9 (82 %)	10 (100 %)	10 (91 %)	7 (70 %)	4 (80 %)
Good LV (>50 %)	11 (100 %)	10 (100 %)	11 (100 %)	10 (100 %)	5 (100 %)
eGFR >55 mL/min	11 (100 %)	10 (100 %)	11 (100 %)	10 (100 %)	5 (100 %)
Rhythm					
Sinus	11 (100 %)	10 (100 %)	11 (100 %)	10 (100 %)	5 (100 %)
Surgery					
CABG	7 (64 %)	4 (40 %)	7 (64 %)	5 (50 %)	1 (20 %)
AVR	4 (36 %)	5 (50 %)	0 (0 %)	5 (50 %)	4 (80 %)
CABG + AVR	0 (0 %)	1 (10 %)	4 (36 %)	0 (0 %)	0 (0 %)
Medications					
β-blocker	8 (73 %)	4 (40 %)	7 (64 %)	2 (20 %)	1 (20 %)
ACE inhibitor	5 (45 %)	5 (50 %)	5 (45 %)	3 (30 %)	1 (20 %)
Calcium channel blocker	2 (18 %)	0 (0 %)	1 (9 %)	1 (10 %)	1 (20 %)
Nitrate	2 (18 %)	2 (20 %)	1 (9 %)	1 (10 %)	1 (20 %)
Statin	8 (73 %)	2 (20 %)	7 (64 %)	5 (50 %)	3 (60 %)
MRA	1 (9 %)	1 (10 %)	0 (0 %)	0 (0 %)	0 (0 %)
Diuretic	2 (18 %)	1 (10 %)	1 (9 %)	0 (0 %)	0 (0 %)
Anti-arrhythmic	0 (0 %)	0 (0 %)	0 (0 %)	0 (0 %)	0 (0 %)
Trabecular dimensions					
Length (mm)	4.31	5.16	5.05	3.86	4.6
Diameter (mm)	0.97	1.01	1.09	1.18	0.89

LV, left ventricle; eGFR, estimated glomerular filtration rate; CABG, coronary artery bypass graft surgery; AVR, aortic valve replacement; ACE, angiotensin converting enzmye; MRA, mineralocorticoid receptor antagonist

*Data expressed as number (%) or mean

Trabeculae were randomly allocated to [1] control (*n* = 11), [2] hypoxic preconditioning (*n* = 10), [3] SDF-1α pre-treatment (*n* = 11), [4] AMD3100 + SDF-1α pre-treatment (*n* = 10), and [5] AMD3100 pre-treatment (*n* = 5). Two separate trabeculae were collected for immunofluorescent staining. The sample was placed in ice-cold buffer prior to careful dissection of the trabeculae. Isolated trabeculae (≤1.2 mm in diameter and ≥2.0 mm in length) were suspended in a heated (37 °C) organ bath with one end connected to a force transducer. Samples were superfused with oxygenated modified Tyrode’s buffer (95 % O_2_/5 % CO_2_) at 37 ± 0.5 °C and pH 7.4 ± 0.5 [5]. The muscle was electrically paced at 1 Hz and stretched until the maximum force of contraction (the peak of the Frank-Starling curve) was achieved. The muscle was subsequently allowed to stabilise for 90 min before being subjected to 60 min of hypoxia by superfusion with equiosmolar, glucose-free hypoxic modified Tyrode’s buffer (95 % N_2_/5 % CO_2_), pH 7.4 ± 0.5 and electrical stimulation at 3 Hz. The muscle was reoxygenated for 60 min with normoxic buffer and 1 Hz stimulation, to simulate reperfusion. Hypoxic preconditioning, consisting of 4.5 min hypoxia and pacing at 3 Hz followed by 6 min reoxygenation and pacing at 1 Hz, was applied immediately prior to the index hypoxic period as a positive cardioprotective control [5]. SDF-1α (25 ng/ml), AMD3100 (10 μg/ml) or saline vehicle were administered for 30 min and 40 min respectively prior to index hypoxia, concentrations that were based on previous publications [4, 5].

### Immunohistochemistry

In a separate group of experiments, isolated human atrial trabeculae were frozen and mounted in OCT before being cut into 5 μm sections at −20°C in a microtome-cryostat and transferred to slides. Sections were fixed with HistoChoice (Sigma-Aldrich, UK) for 20 min at room temperature and washed with PBS, before blocking with 5 % BSA/PBS for 60 min. Immunofluorescent co-staining of CXCR4 and cardiomyocytes was performed using rabbit monoclonal anti-CXCR4 (ab124824) and mouse anti-cardiac troponin T (ab8295) from Abcam (Gillingham, UK). Anti-rabbit Alexa Fluor 488 and anti-mouse Alexa Fluor 555 secondary antibodies were purchased from Abcam. Cardiomyocyte co-stained samples were incubated in anti-CXCR4 and anti-cardiac troponin T, diluted in 1 % BSA/PBS 1:100 and 1:10 respectively, overnight at 4°C. Following washing and incubation with the appropriate secondary antibody diluted 1:400 in the same buffer for 60 min at room temperature, samples were washed again and coverslips mounted using fluorescence mounting medium (Dako, Ely, UK). 0.1 μg/ml Hoechst 33,258 nuclear stain (Life Technologies, Paisley, UK) was added with the secondary antibodies to all sections. Preparation of control sections was identical and they were incubated either with 1 % BSA/PBS only (unstained control) or with the relevant secondary antibody in the absence of any primary antibody. After drying, Alexa 488 and Alexa 555 fluorescence was imaged using a 40× oil immersion objective, by sequential scanning using the 488 nm and 543 nm lines of a Leica SP5 confocal microscope and collecting emitted light at 500–530 nm and 580–650 nm respectively. Control experiments were performed to confirm the absence of fluorescence bleed-through or non-specific staining with secondary antibodies alone.

### Statistics

The final force of contraction in the human atrial trabeculae hypoxia-reoxygenation experiments was expressed as a percentage of baseline contractility to give recovery of function. Values are expressed as mean ± SEM. Comparisons between more than 2 groups were made using 1-way analysis of variance (ANOVA). Fisher’s protected least significant difference post hoc test was used for between-group comparisons. Differences were considered statistically significant when *P* < 0.05.

## Results

**Fig. 1:**
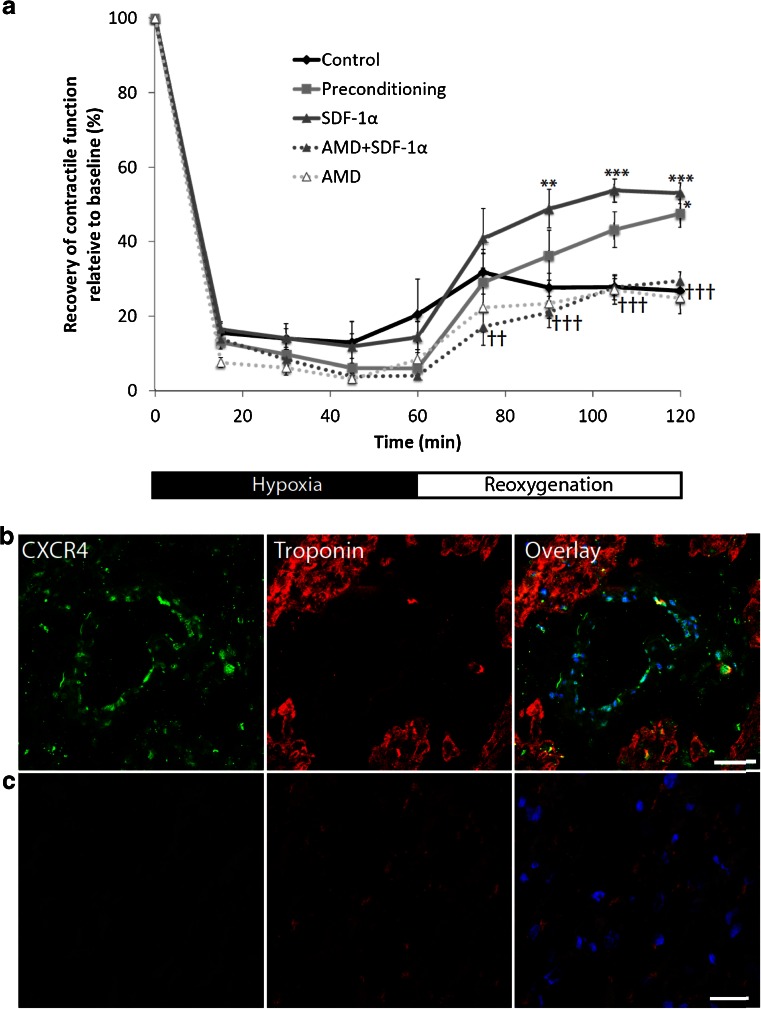
The SDF-1α-CXCR4 axis protects human myocardium from hypoxia-reoxygenation injury: **a** Recovery of contractile function during simulated ischaemia and reperfusion applied to isolated human atrial trabeculae is improved by pre-treatment with exogenous SDF-1α to a level similar to that conferred by hypoxic preconditioning. N = 11 atrial trabeculae in the control and SDF-1α pre-treatment groups, 10 in the hypoxic preconditioning and AMD3100 + SDF-1α pre-treatment groups, and 5 in the AMD3100 pre-treatment group. *SDF-1α vs. control, †AMD + SDF-1α vs. SDF-1α (*P < 0.05, **P < 0.005, ***P < 0.001). **b**, Immunofluorescent staining of isolated human atrial trabeculae demonstrating the distribution of CXCR4 (green) in relation to cardiomyocytes (red). **c**, Immunofluorescent staining using secondary antibody in the absence of primary antibody. Representative images from N = 2 independent experiments, 50 μm scale bar

In control trabeculae, the mean recovery of function after hypoxia and reoxygenation was 27 ± 2 % [1]. Recovery of function in samples pre-treated with SDF-1α was significantly increased (53 ± 3 %, *P* < 0.05 vs. control, Fig. 1), which was similar to that in control trabeculae subjected to hypoxic preconditioning (48 ± 4 %, *P* < 0.05 vs. control).

The role of signalling via CXCR4 was investigated by pre-treating trabeculae with the specific CXCR4 inhibitor, AMD3100, which abrogated the protective effect of SDF-1ɑ (30 ± 2 %, *P* < 0.05 vs. SDF-1α alone). AMD3100 alone had no effect on functional recovery (25 ± 4 %). Furthermore, immunofluorescent staining confirmed the presence of CXCR4 receptors on cardiomyocytes in human atrial trabeculae (Fig. 1).

## Discussion

SDF-1α is a small CXC chemokine that is expressed by several organs and tissues, including endothelial cells and cardiomyocytes in the heart [6]. Its cognate receptors CXCR4 and CXCR7 are similarly present in a range of tissues, including cardiomyocytes. The SDF-1α/CXCR4 axis has garnered considerable interest due to its role in stem cell homing, angiogenesis and ventricular remodelling after myocardial infarction, and has been used to target stem cells to ischaemic tissue, thereby improving LV dimensions and function [7].

We and others have previously demonstrated a cardioprotective role for SDF-1α on both infarct size in isolated perfused rat hearts and recovery of function in isolated rat papillary muscle [5, 8]. Both of these beneficial effects were abolished by AMD3100, all of which evidences a cardioprotective role for the SDF-1α/CXCR4 axis. Elsewhere, the application of exogenous SDF-1ɑ has been shown to reduce apoptosis in isolated cardiomyocytes and SDF-1ɑ infused into the murine LV cavity in vivo produced significantly smaller infarct sizes after IRI, both effects that were abrogated by AMD3100 [9].

This ex vivo functional model of hypoxia and reoxygenation translates the aforementioned findings to human myocardium for the first time, in the native milieu of the functional heart. This study confirms [1] the ability of exogenous SDF-1α to protect human atrial trabeculae from the detrimental effects of simulated IRI; [2] the role of CXCR4 in this mechanism, as evidenced by attenuating the benefits of SDF-1α using the specific receptor blocker AMD3100; and [3] the presence and distribution of CXCR4 in human adult cardiomyocytes and endothelial cells.

There is evidence that the cardioprotective utility of SDF-1α/CXCR4 may be conferred by activating intracellular pro-survival kinases, although the specific mechanism remains unknown [5]. However, it is thought to converge on the mitochondrial permeability transition pore (mPTP) and effect protection by delaying its opening and consequent necrotic cell death [10]. The exact mechanism of protection afforded by SDF-1α/CXCR4 should be the focus of further investigation in both basic and clinical studies.

In summary, our findings support the hypothesis that exogenous SDF-1α is a preconditioning mimetic, herein shown to exert acute protection against the deleterious effects of hypoxia and reoxygenation and that this protection, in the isolated human atrial trabeculae, occurs via the CXCR4 receptor.
